# Temperature-mediated phase transformation, pore geometry and pore hysteresis transformation of borohydride derived in-born porous zirconium hydroxide nanopowders

**DOI:** 10.1038/srep26404

**Published:** 2016-05-20

**Authors:** Nadiya B. Nayak, Bibhuti B. Nayak

**Affiliations:** 1 Department of Ceramic Engineering, National Institute of Technology Rourkela, Odisha 769 008, India

## Abstract

Development of in-born porous nature of zirconium hydroxide nanopowders through a facile hydrogen (H_2_) gas-bubbles assisted borohydride synthesis route using sodium borohydride (NaBH_4_) and novel information on the temperature-mediated phase transformation, pore geometry as well as pore hysteresis transformation of in-born porous zirconium hydroxide nanopowders with the help of X-ray diffraction (XRD), Brunauer–Emmett–Teller (BET) isotherm and Transmission Electron Microscopy (TEM) images are the main theme of this research work. Without any surfactants or pore forming agents, the borohydride derived amorphous nature of porous powders was stable up to 500 °C and then the seed crystals start to develop within the loose amorphous matrix and trapping the inter-particulate voids, which led to develop the porous nature of tetragonal zirconium oxide at 600 °C and further sustain this porous nature as well as tetragonal phase of zirconium oxide up to 800 °C. The novel hydrogen (H_2_) gas-bubbles assisted borohydride synthesis route led to develop thermally stable porous zirconium hydroxide/oxide nanopowders with an adequate pore size, pore volume, and surface area and thus these porous materials are further suggested for promising use in different areas of applications.

Zirconium oxide and zirconium oxide-based materials have been the subject of intense research because the materials promise extensive use in catalysts or catalytic supports^1^,^2^ optical waveguides^3^, gate dielectric^4^, high-performance ceramics^5^, biological materials^6^, and wide gap band semiconductor^3^. Generally, zirconium hydroxide in the amorphous state was developed in the as-synthesized condition, when it was derived via wet-chemical synthesis routes using the common precipitating agent such as NH_4_OH^7^,^8^. During calcination process, amorphous zirconium hydroxide may develop either cubic (c) or tetragonal (t) or monoclinic (m) or mixture of ‘c’ or ‘t’ or ‘m’ phases of zirconium oxide depending on various optimization parameters^9^,^10^,^11^,^12^. Zirconium oxide in the form of cubic (c) or tetragonal (t) at room temperature was considered to be an important ceramic material. In the same time, it was difficult to develop stable phase of c/t-zirconium oxide up to moderate temperature unless until controlling the size of zirconium hydroxide/oxide during synthesis/calcination. According to Gravie, pure t-zirconium oxide is stable up to a certain critical size of 30 nm^13^. However, the nuclei of zirconium hydroxide grow in rapid way through agglomeration, when using without adding any surfactants or stabilizers. So, this is a great challenge to retain t-zirconium oxide within this critical size at moderate temperature. In addition, fabrication of porous oxide materials with different pore morphology has an important role in addressing some of the shape and size selective potential applications^14^. The shape and size of the pores and their distribution directly affect their ability to function in a particular application^15^,^16^,^17^,^18^,^19^,^20^. Porous zirconium oxide with precisely designed pore structures has found to be promising application for catalyst supports, heat insulation, particle filters and gas membranes under severe conditions such as high-temperature and corrosive environments^17^,^21^,^22^,^23^. Porous zirconium oxide microspheres with unique pore structure and narrow size distribution were found to be suitable for high separation efficiency^20^. Crystalline nature of porous tetragonal zirconium oxide with large pores was applicable for catalysis application^24^. In addition, porous amorphous zirconium hydroxide/oxide has found to be promising application for removal of toxic ions^17^. In this context, different synthesis methods including gelation, precipitation, hydrothermal and sonochemical using the common surfactant-assisted template method were accommodated to prepare porous materials with various shape and size^25^,^26^,^27^. However, this process of template method is inconvenient and damages the desired configurations of pores at lower or moderate temperature during the removal of the templates^28^,^29^. In addition, the above syntheses derived porous zirconium oxide shows poor structural stability (i.e. phase transformation of zirconium oxide from tetragonal to monoclinic (m), when the sample was cooled down from high or moderate temperature to ambient condition) as well as collapse of porous structure at lower or moderate temperature and thus affect the potential application of porous zirconium oxide^28^,^30^. So, gas bubbles-assisted synthesis strategy knocks the door of research to short out the problem of surfactant template-assisted synthesis. This gas-bubbles template route is a novel, convenient, environmental friendly as well as effective method to develop porous nanoparticles in modern research^31^. Up to date, CO_2_, H_2_S, NH_3_, N_2_ gas bubbles are employed to create gas-bubbles aggregation center for the development of various porous metal oxides^32^,^33^,^34^,^35^. In this perspective, our group have explored H_2_ gas-bubbles evolving aqueous sodium borohydride (NaBH_4_) to develop zirconium oxide, a transition metal oxide via borohydride synthesis route using gelation, precipitation and constant pH method^36^. The constant pH mode of synthesis for the development of porous zirconium oxide via borohydride route is a new strategy. In aqueous state, the NaBH_4_ reacts vigorously with water molecules and form two active species such as tetrahydroxy borate ion B(OH)_4_^−^ ions and along with huge amount of H_2_ gas bubbles via constant pH process^36^. The formation of B(OH)_4_^−^ ions in solution helps to form a precipitate form of zirconium hydroxide [Zr(OH)_4_] nuclei and the H_2_ gases generated in the precursor solution were released as gas-bubbles, which act as free-templates and thus these bubbles creates numerous gas-liquid interface inside the solution. The gas–liquid interfaces in the solution may serve as the nucleation or agglomeration centers throughout the solution^37^. Since the system was surfactant-free, the highly energetic Zr(OH)_4_ nuclei was not protected with foreign species like surfactants, so the nuclei had the tendency to aggregate together to release the high surface energy. The formation of the bubbles in the reaction system may enable this agglomeration process to proceed in a controllable way. With the introduction of bubbles into the reaction system, the agglomeration process occurred around the bubbles. Further, these gas-bubbles may create interconnected voids due to escaping of gas-bubbles in dry state. So, the particles of as-synthesized powder may be well separated with each other by voids/pores. This loose nature of in-born nanopowders of zirconium hydroxide may lead to develop porous zirconium oxide with various pore morphologies during calcination process. Additionally, it may also help to develop and stable both the amorphous as well as tetragonal zirconium oxide up to moderate temperatures, so that it may be suitable for various possible applications within a wide temperature range. So, in this current research work, the main motivations are to (i) analyze the phases present during calcination of borohydride derived as-synthesized powders, (ii) retain both amorphous as well as crystalline phase (t) of zirconium oxide up to moderate temperature, (iii) retain porous nature in amorphous as well as crystalline powders up to moderate temperature, (iv) study the nature of porous nanoparticles in terms of pore size, shape and its distribution at different calcination temperature, (v) understand the pore hysteresis of the as-synthesized as well as calcined samples, and (vi) correlate the pore size, pore volume as well as surface area of borohydride derived porous powders with the available literatures to find out the potential of these materials for use in different applications.

## Results and Discussion

**Figure 1:**
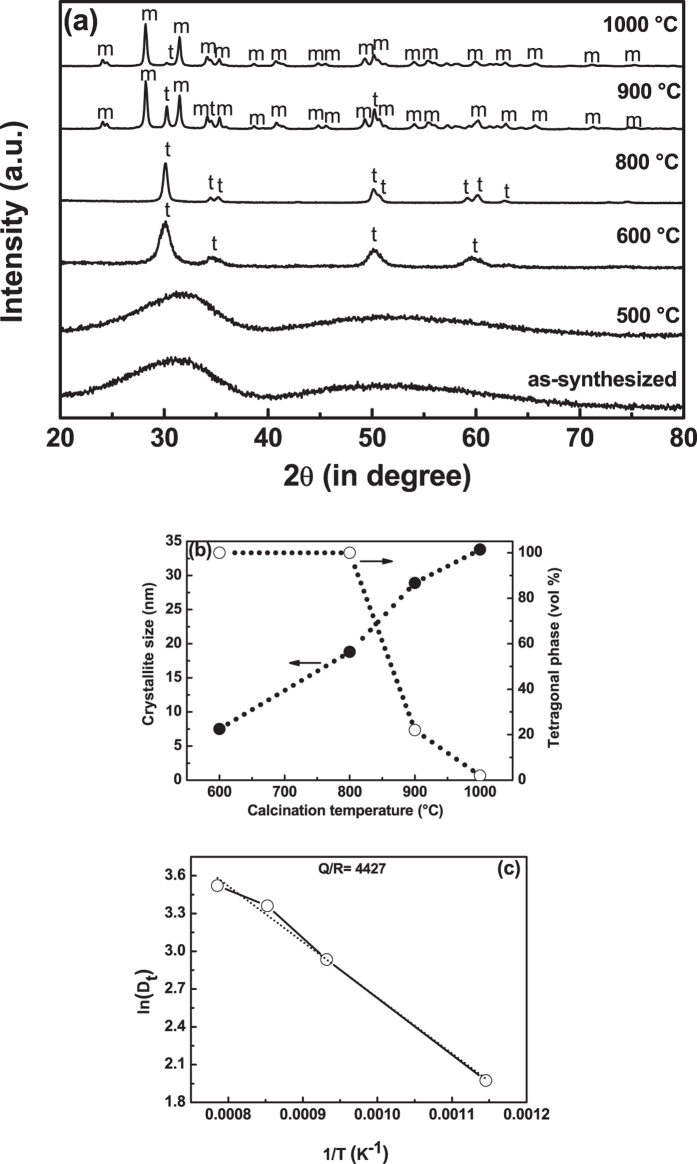
X-ray diffraction pattern of the as-synthesized as well as calcined samples (**a**) [Note: ‘t’ stands for tetragonal and ‘m’ stands for monoclinic zirconium oxide] and crystallite size and volume percentage of tetragonal phase as a function of calcination temperature (**b**). To determine the activation energy (Q) of particle growth, Fig. 1(**b**)was re-plotted to Fig. 1(**c**).

**Figure 2:**
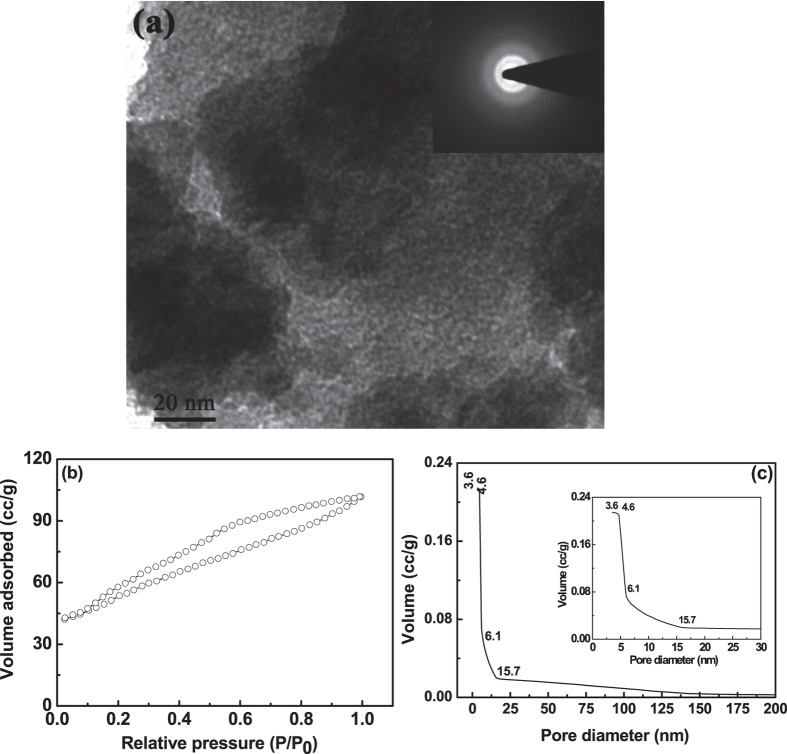
TEM micrograph (**a**) of as-synthesized porous zirconium hydroxide and the corresponding electron diffraction pattern in the inset of (**a**) indicates that the powder was amorphous in nature. BET-isotherm (**b**) and pore size distribution (**c**) of the as-synthesized porous zirconium hydroxide. Inset of (**c**) is the enlarge view of the pore size distribution.

The phase analysis was performed by X-ray diffraction and Fig. 1 shows X-ray diffraction patterns of borohydride derived as-syntesized and calcined powders. The amorphous nature of borohydride derived as-synthesized powders remain amorphous up to 500 °C and further it slowly converts to crystalline in nature at 600 °C. All the peaks of powder calcined at 600 °C are identified and indexed with tetragonal form of zirconium oxide (t-zirconium oxide), as per JCPDS file no: 79–1768. Further, the tetragonal nature of zirconium oxide was found to be stable up to 800 °C. A mixture phases of t-zirconium oxide (78 vol%) and monoclinic (m) phase [peaks are indexed as per JCPDS file no: 83–0943] of zirconium oxide (22 vol%) were present in the sample, calcined at 900 °C. At further higher calcination temperature of 1000 °C, major phase of m-zirconium oxide (98 vol%) along with minute amount of t-zirconium oxide (2 vol%) were observed. The growth of crystallite size of zirconium oxide was strongly affected by the thermal treatment and also helps for stabilizing t-zirconium oxide up to 800 °C. Further, the crystallite size and volume percentage of t-zirconium oxide as a function of calcination temperature was represented in Fig. 1(b). It was found that the crystallite size increases with calcination temperature, but at a slower rate and thus it may help to stable the t-zirconium oxide up to 800 °C. Further, the computed values of the crystallite size of t-zirconium oxide powders calcined at 600 °C to 1000 °C enabled the calculation of the activation energy of particle growth (Q, kJ/mol) of t-zirconium oxide phase using Arrhenius equation: D_t_ = Ae^(−Q/RT)^, where D_t_ denote the crystallite size (nm) calcined at temperature T, A is the frequency factor of Arrhenius equation, and Q is the activation energy of particle growth, R is the gas constant (J/mol.K) and T is the calcination temperature (K)^38^. To determine the activation energy (Q) of particle growth, Fig. 1(b) was re-plotted to Fig. 1(c), assuming that crystallite growth in nanopowders of zirconium oxide, being a thermally activated process and is dependent on the calcination temperature. The activation energy of particle growth of borohydride synthesized zirconium oxide was found to be ~36.8 kJ/mol, which is much lower than that of yttria-stabilized zirconium oxide, but similar to that for pure nano zirconium oxide^39^. The slow growth of the particles during calcination process led to stable the amorphous nature up to 500 °C and also stables the t-zirconium oxide up to 800 °C. Additionally, the nature of powder morphology of as-synthesized sample may also play a role for stabilizing the amorphous phase up to 500 °C and t-zirconium oxide up to 800 °C. So, powder morphology was performed using TEM and the TEM image of the as-synthesized powders was shown in Fig. 2(a).

The as-synthesized powders are found to be amorphous in nature, as it shows hazy rings of electron diffraction pattern [inset of Fig. 2(a)]. The amorphous powders were found to be loose nature, as observed from TEM micrograph of Fig. 2(a) and it indicates that the individual fine particles are well-separated by voids/pores. It was also observed that the nature of pores in the as-synthesized condition seems to be interconnected. To further justify the pore geometry, BET adsorption-desorption isotherm was performed on the as-synthesized powders and was shown in Fig. 2(b). The existence of wide broad hysteresis loop in BET-isotherm indicates that the in-born as-synthesized powders are porous in nature and was well correlated with TEM micrograph [Fig. 2(a)]. Further, the type of pore was analyzed from the BET hysteresis loop. The wide hysteresis loop also indicate a delay in both condensation and evaporation process^19^. In this BET-isotherm, the adsorption follows a slow increase in adsorbed volume with increases in partial pressure without any saturation point, but with a slight slope change at P/P_0_ at 0.6. However, desorption curve follows a different path forming a wide hysteresis loop of type H2 (according to IUPAC classification) with a slight slope change at similar P/P_0_ of 0.6 and closes at the starting point of adsorption. This H2 type hysteresis loop indicates the existence of ink bottle-neck type pores in the gas-bubbles derived as-synthesized zirconium hydroxide powders^40^,^41^. Further, due to non-saturating behavior of adsorption curve and delay in desorption curve along with the closure point at P/P_0_ of 0.1 indicates that these ink bottle-neck pores are well interconnected in nature^16^,^41^,^42^. Analyzing the hysteresis loop by considering the ink bottle-neck type pores, it was further suggests that the first half of the BET adsorption isotherm (up to P/P_0_ at 0.6) represents the condensation of surface necks and second half of this adsorption isotherm associated with continuous condensation of the inner interconnected bottles through narrow necks without a saturation point^43^. During desorption process, first half (up to P/P_0_ at 0.6) represents the evaporation from surface necks of all bottles and second half of desorption closes at P/P_0 _~ 0.1 is thought to depend not only on size of the bottles, but also on the connectivity of the pore network^42^. The interconnected network nature of ink bottle-neck pores gives rise to pore-blocking effects, which also delays both adsorption and desorption mechanism^43^. The pore size distribution of as-synthesized porous zirconium oxide was also calculated from BJH desorption curve and was shown in Fig. 2(c). The highest pore volume was found to be ~0.21 cc/g for the pore diameter of 3.6 nm to 4.6 nm. However, the pore diameter of as-synthesized zirconium hydroxide varies from 3.6 nm to 15.7 nm (see enlarge view of pore size distribution in the inset of Fig. 2(c)]. So, it seems that the pore size of as-synthesized zirconium hydroxide was fall in the meso-range (2 to 50 nm), but may also contain some amount of micro pores (<2 nm), as the volume content of minimum pore size (3.6 nm) was same as that of 4.6 nm^44^. In addition, the surface area of as-synthesized zirconium hydroxide powders was found to be 182 m^2^/g.

**Figure 3:**
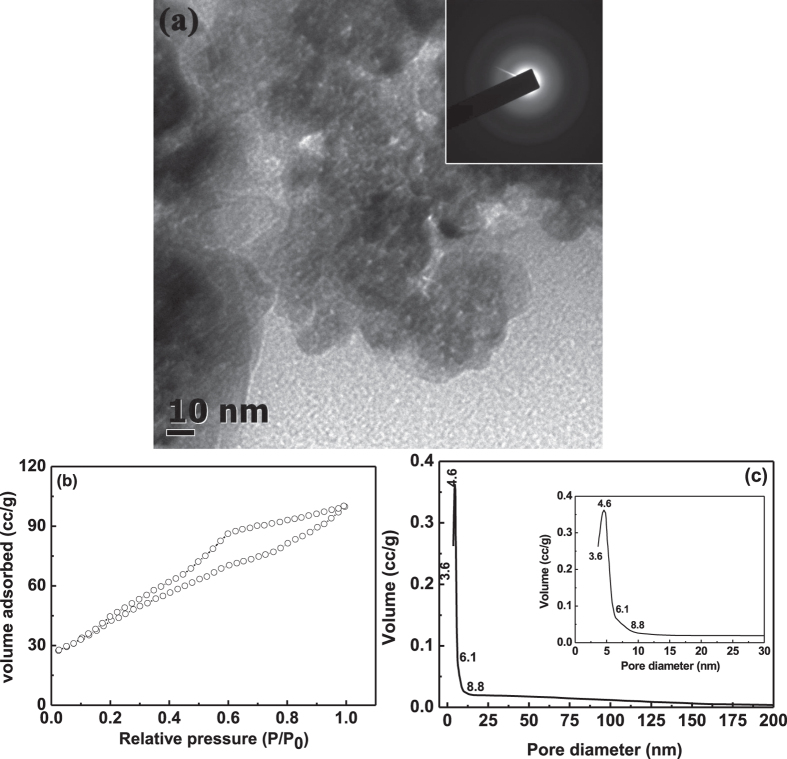
TEM micrograph (**a**) of the as-synthesized powders calcined at 500 °C and the corresponding electron diffraction pattern in the inset of (**a**) indicates that the powder was amorphous in nature. BET-isotherm (**b**) and pore size distribution (**c**) of the calcined (500 °C) porous powders. Inset of (**c**) is the enlarge view of the pore size distribution.

The pore geometry of the in-born interconnected loose porous nanoparticles may strongly modify the pore morphology of powders during calcination process. So, the loose porous as-synthesized powders were further calcined at different temperatures. Figure 3(a) shows TEM micrograph of porous as-synthesized powders calcined at 500 °C. Up to this temperature, the amorphous nature still remains same, as confirmed from the hazy rings of electron diffraction pattern, indicated in the inset of Fig. 3(a). TEM micrograph of Fig. 3(a) clearly indicates that the powders calcined at 500 °C are still porous in nature. The presence of intra-particle voids may inhibit the mass transfer between loose nanoparticles and also help to restrict the coarsening of particles, during calcination up to 500 °C. So, formation of smaller sized particles with interconnected voids along with lower activation energy of particle growth during calcination process led to sustain the amorphous phase of up to 500 °C. BET adsorption-desorption isotherm was performed on this calcined powders and was shown in Fig. 3(b). The nature of BET-isotherm was found to be same of H2 type as that of as-synthesized powders. In this isotherm, the high pressure unsaturated adsorption and a comparable change in the slope at P/P_0_ = 0.6 in desorption curve with a slightly higher closure point at P/P_0_ = 0.2 (as compared to desorption behavior of as-synthesized sample) reflects that the pores are well interconnected, but the inter-connection are blocked due to neck closing of a very few bottle-neck pores during calcination process and thus leads to easy evaporation of condensed gas during desorption process^45^. The pore size distribution was evaluated from BJH desorption curve and was shown in Fig. 3(c). The pore size distribution of this sample was becoming narrow [see enlarge view of pore size distribution in the inset of Fig. 3(c)] as compared to pore size distribution of as-synthesized sample, which further suggests that this calcined powders was purely mesoporous in nature, as the pore size varies from 3.6 nm to 8.8 nm with as maxima of 4.6 nm. In addition, the pore volume was found to ~0.36 g/cc, which is slightly higher than the pore volume of as-synthesized sample of same particular pore size of 4.6 nm. In the case of as-synthesized sample, the lower volume containing pores having larger diameter in the range between 6.1 nm to 15.7 nm indicates that some of the pores may connect with each other, but without a neck similar to dumb-bell shape. Heating at 500 °C, the size of lower volume containing dumb-bell shaped pores varies in between 6.1 nm to 8.8 nm. The decrease of dumb-bell shaped pores size from 15.7 nm to 8.8 nm at 500 °C was mainly due to the formation of additional bottle-neck pore from dumb-bell shaped pores via coarsening during calcination process. So, the increase in pore volume for particular pore diameter of 4.6 nm was due to conversion of interconnected dumb-bell shaped pores to bottle-neck pores. Additionally, the surface area of the powder calcined at 500 °C was decreased to 160 m^2^/g.

**Figure 4:**
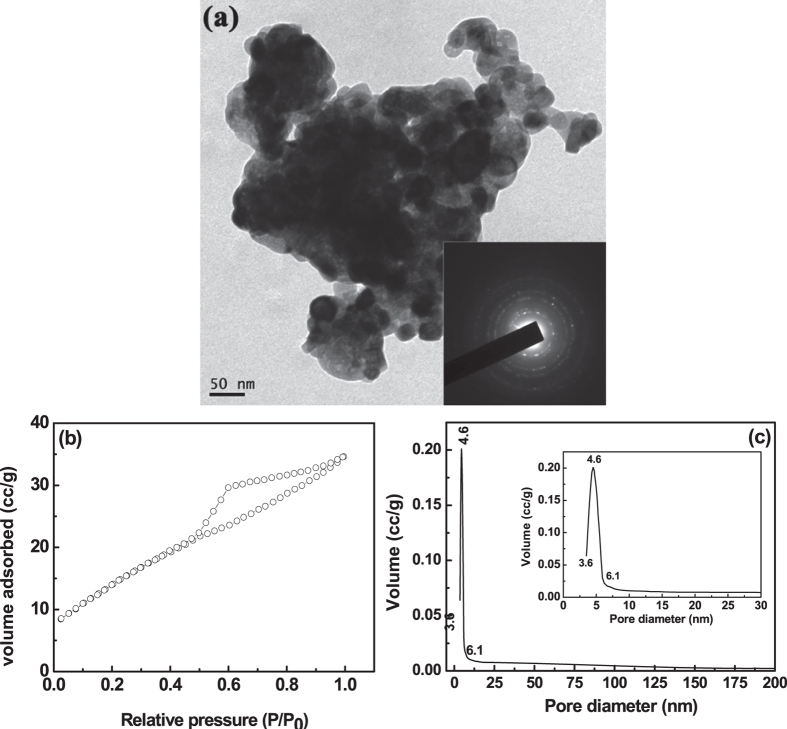
TEM micrograph (**a**) of the as-synthesized powders calcined at 600 °C and the corresponding electron diffraction pattern in the inset of (**a**) indicates that the powder was in crystalline in nature. BET-isotherm (**b**) and pore size distribution (**c**) of the calcined (600 °C) porous zirconium oxide. Inset of (**c**) is the enlarge view of the pore size distribution.

Figure 4(a) shows TEM micrograph of the powders calcined at 600 °C and the electron diffraction pattern was indicated in the inset of Fig. 4(a). Hazy dotted ring pattern of electron diffraction indicates that the amorphous nature was not fully converted to crystalline nature of t-zirconium oxide at this temperature. While increase in calcination temperature, the seed crystals start to develop within the loose amorphous matrix and these nanocrystals grow as well as traps the inter-particulate voids and form a trapped pore within the particle. So, pores are entrapped within individual zirconium oxide particles and developed porous structure. The pore morphology of this crystalline t-zirconium oxide was analyzed using BET-isotherm and was shown in Fig. 4(b). In this BET-isotherm, adsorption curve increases with increase in partial pressure with non-saturating behavior and forming a hysteresis loop with the help of desorption curve. This desorption curve suddenly drops at P/P_0_ = 0.6 and matches with the adsorption curve at P/P_0_ = 0.5. The curvature of BET hysteresis loop is typically H2 type indicating ink bottle-neck type pores and some pores are interconnected due to non-saturating behavior of adsorption curve. The pore size distribution was evaluated from BJH desorption curve and was shown in Fig. 4(c). The pore size distribution was becoming still narrow [see enlarge view of pore size distribution in the inset of Fig. 4(c)] as compared to pore size distribution of 500 °C heated sample, but the pore size varies from 3.6 nm to 6.1 nm with as maxima of 4.6 nm. It also further indicates that with increase in calcination temperature from 500 °C to 600 °C, the mesoporosity remain same in porous t-zirconium oxide. But, the pore volume of the particular diameter of 4.6 nm decreases to 0.20 g/cc at 600 °C and this may be due to coarsening of nanoporous particles. The decrease in pore volume with narrow size distribution (as compared to BET-isotherm of 500 °C heated sample) leads easy evaporation of condensed gas and thus shifts the closure point of desorption branch at P/P_0_ = 0.5. The surface area of crystalline t-zirconium oxide further decreases to 55 m^2^/g at 600 °C.

**Figure 5:**
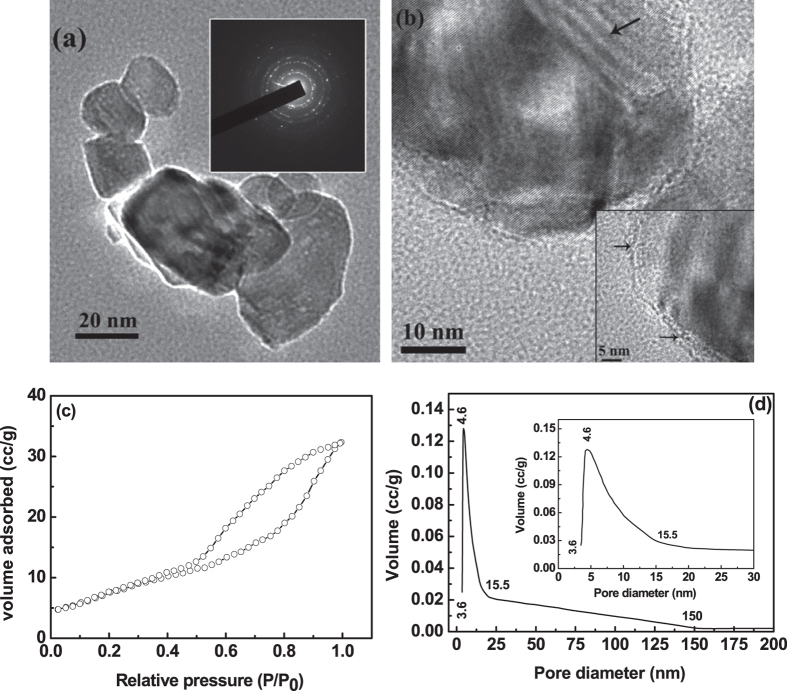
TEM micrograph (**a**) of the as-synthesized powders calcined at 800 °C and the corresponding electron diffraction pattern in the inset of (**a**) indicates that the powder was in crystalline in nature. Higher magnified TEM image (**b**) indicates the presence of two types (spherical as well as lamellar) of pore. Inset of (**b**) indicates the presence of ultra-fine loose particles on the surface of the particle. BET-isotherm (**c**) and pore size distribution (**d**) of calcined (800 °C) zirconium oxide. Inset of (**d**) is the enlarge view of the pore size distribution.

Further, TEM was performed on 800 °C heated sample in order to study the particle morphology and pore geometry of t-zirconium oxide. Figure 5(a) shows TEM micrograph of calcined (800 °C) t-zirconium oxide and the electron diffraction pattern was indicated in the inset of Fig. 5(a). Sharp dotted ring pattern of electron diffraction indicates that the zirconium oxide was purely crystalline in nature. At this temperature, particle shape is nearly spherical to polyhedral in shape. The sizes of these particles are in the range between ~20 to ~40 nm. In each particle, the fine pores are well distributed as well as the surface of the particle seems to be in different morphology, as observed from TEM micrograph in Fig. 5(a). In addition, the coarsening of fine particles along with coalescence of existing pores take place at higher temperature and develop a stable phase of porous t-zirconium oxide at 800 °C. To justify the pore as well as surface morphology of t-zirconium oxide, a higher magnified TEM was analyzed and shown in Fig. 5(b). It was confirmed that the zirconium oxide particle consists of two different types of pore geometry such as nearly spherical and lamellar type [marked as arrow in Fig. 5(b)]. The size of spherical pores varies from ~3 nm to ~12 nm and the thickness of lamellar type pores was ~3 nm. It was also observed that a thin disordered layer was covering each particles and this layer was visualized in a clear way, as shown in the inset of Fig. 5(b). This thin layer consists of ultra-fine loose particles [marked as arrow in the inset of Fig. 5(b)] with a thickness of around 2 to 4 nm. The pore morphology of calcined (800 °C) t-zirconium oxide was also analyzed using BET-isotherm and was shown in Fig. 5(c). The presence of hysteresis loop indicates that the t-zirconium oxide particles retain their porous nature up to 800 °C. However, the behavior of hysteresis loop was quite different from the hysteresis behavior of lower temperature calcined samples. This hysteresis loop is typically consists of mixture of ink bottle-neck and slit type pores (H2 + H3 type). At this temperature, the pore hysteresis transformed from H2 type to mixed H2 and H3 type and was also well-correlated with TEM micrographs. It was well understood that the coarsening of porous particles having lower diameter (~3.6 nm) bottle-neck pores is faster than the higher diameter bottle-neck pores at 800 °C. At the same time, the entrapped air in pores expands at this temperature and migrates towards the surface with high pressure without closing the interconnected paths and thus forming a thin layer of ultra-fine loose particles on the surface of t-zirconium oxide. Further, the lower diameter pore diffuses and matches with the interconnected paths and thus creating lamellar type, whereas the higher diameter pore remains as bottle-neck type pore. The pore size distribution was evaluated from BJH desorption curve and was shown in Fig. 5(d). From this figures, it was found that the pore size varies from 3.6 nm to 150 nm with a maximum of 4.6 nm with wide pore size distribution [see enlarge view of pore size distribution in the inset of Fig. 5(d)]. In this t-zirconium oxide sample, the pores are mostly mesoporous in nature along with some lower amount of macro porous in the range between 50 nm to 150 nm. Additionally, the wider distribution was mainly due to the additional contribution of lamellar type pores. High temperature coarsening of particles leads to decrease the bottle-neck pore volume of the particular diameter of 4.6 nm to 0.129 g/cc. In addition, the surface area of porous zirconium oxide, calcined at 800 °C was found to be ~29 m^2^/g.

**Figure 6:**
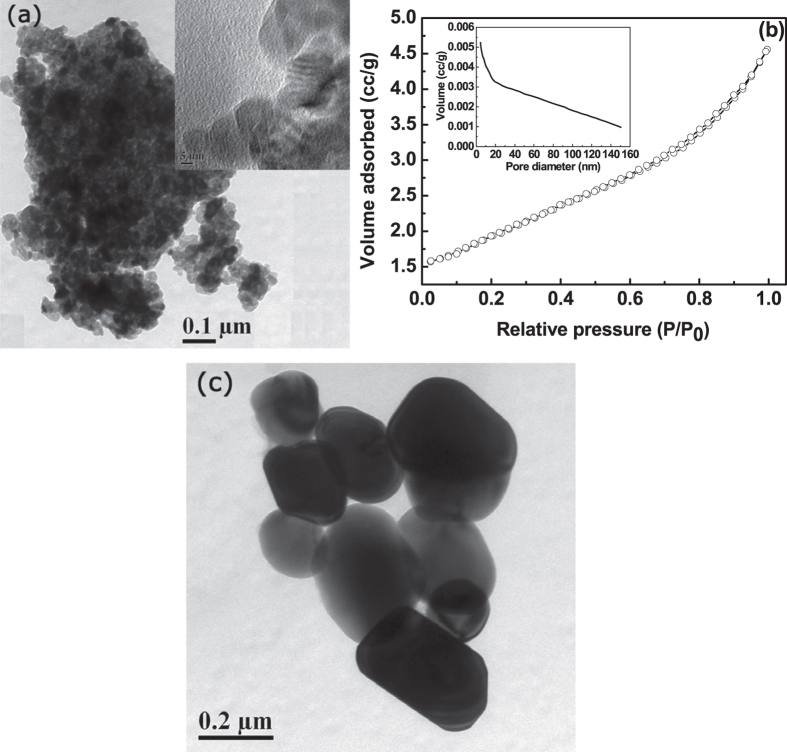
TEM micrograph (**a**) of the as-synthesized powders calcined at 900 °C. BET-isotherm (**b**) and inset of (**b**) shows the pore size distribution. TEM micrograph (**c**) indicates the non-porous zirconium oxide, calcined at 1000 °C.

At still higher temperature of 900 °C, the particle morphology of zirconium oxide was studied using TEM micrograph and was shown in Fig. 6(a). The powder morphology was seem to be porous in nature, however, from inset of Fig. 6(a), it was observed that the calcined (900 °C) zirconium oxide contain lamellar type pores. BET-isotherm in Fig. 6(b) also confirms that the pore morphology was typically lamellar of H3 type. So, at 900 °C, remaining bottle-neck pores diffuse with the interconnected paths and forming lamellar type. The average pore diameter decreases from 4.6 nm to 3.6 nm at 900 °C and pore volume also decrease to 0.005 cc/g. The surface area of zirconium oxide calcined at 900 °C was found to be 6 m^2^/g. However, at higher temperature of 1000 °C, all the pores were diffused completely and forming a fully non-porous spherical and polyhedral particles having size ranges from 150 nm to 400 nm, as confirmed from TEM micrograph of Fig. 6(c).

Based on the XRD, BET-isotherm and TEM image analysis, the H_2_ gas-bubbles assisted borohydride route was found to be a potential synthesis method for development of thermally stable porous nanopowders of zirconium hydroxide or oxide having adequate pore size, pore volume and surface area within a temperature range from 500 °C to 800 °C. More importantly, these porous nanopowders can be used efficiently in different areas of applications. So, in this context, it is justified to compare the textural properties of borohydride derived as-synthesized as well as calcined porous nanopowders with the available reported literatures. Further, Table 1 summarizes the textural properties of the borohydride derived as-synthesized as well as calcined porous nanopowders. Moreover, the phase stability, pore size, pore size distribution and surface area are the important parameters to find out the potential use of these zirconium hydroxide or oxide nanopowders in various fields such as adsorption of heavy metal ions, catalytic reaction, oxygen sensors, storage of gases and luminescent applications^4^,^21^,^24^,^27^,^46^. In view of the phase stability of t-zirconium oxide up to moderate temperature, many researchers have been successfully synthesized t-zirconium oxide via various synthesis methods using surfactants or additives^47^,^48^,^49^. However, in most of the cases, the retention of porous nature in t-zirconium oxide was limited in the range between 300–600 °C and which further led to prevent the use of these porous powders for specific applications^50^,^51^. In this perspective, without the use of any additives or surfactants, the borohydride route is found to be much more advantageous because of the development of in-born porous nature of amorphous zirconium hydroxide in the as-synthesized condition, stable the amorphous as well as porous structure nature up to 500 °C as well as stable t-zirconium oxide nanopowders with porous structure up to 800 °C. Further, the surface area, mean pore diameter and pore volume of borohydride derived powders calcined at 500 °C was comparable with the calcined (450 °C) amorphous zirconium oxide powders prepared by Cui *et al*.^17^, which was having a surface area of 161.8 m^2^/g, mean pore diameter ~9 nm and pore volume of 0.43 cc/g. It was further suggested that this type of large surface area, high pore volume and loose porous structure may be a strong candidate for heavy metal adsorption for environmental application^20^. Similarly, Kuai *el al*.^52^ have developed disordered pores of various transition metal oxides by surfactant assisted aerosol spray method and it was observed that the amorphous nature of zirconium oxide sample heated at 400 °C having a surface area of 116 m^2^/g and a pore volume of 0.16 cc/g. This type of large surface area and pore volume can be widely used in various fields of applications^21^. In addition, the obtained surface area of porous amorphous powders derived via borohydride route was found to be quite comparable with the literatures based on the synthesis of highly ordered porous powders developed with addition of additives or surfactants^50^,^53^. Also, the pore volume and pore diameter of the borohydride derived porous t-zirconium oxide (calcined at 600 °C) can be well comparable with the pore volume and pore diameter of ordered porous t-zirconium oxide prepared using surfactants^54^. Further, the surface area, mean pore diameter and pore volume of 600 °C calcined t-zirconium oxide can also be well comparable with the disordered structure of porous zirconium oxide prepared using surfactants^55^. The calcined (500 °C) t-zirconium oxide prepared from the thermal decomposition of metal– organic frameworks by Yan *et al*.^56^ shows a pore diameter of 5–8 nm with pore volume of 0.208 cc/g. Similarly, Chen *et al*.^57^ have developed t-zirconium oxide at 500 °C which have a lower surface area of 95 m^2^/g and total pore volume as high as 0.19 m^2^/g. In addition, Mokhtar *et al*.^38^ have prepared t-zirconium oxide (calcined at 600 °C) with using surfactant and achieve a lower surface area of 41 m^2^/g and pore volume of 0.3315 cc/g. Further, the retention of porous nature of t-zirconium oxide up to 800 °C was found to be difficult, while synthesized using surfactant-assisted synthesis routes^7^,^10^,^55^. Based on the above discussion, the borohydride derived porous nature of amorphous as well as crystalline t-zirconium oxide nanopowders can efficiently be used for different applications.

## Conclusions

Interconnected porous structure with loose amorphous nature of zirconium hydroxide was successfully prepared through gas-bubble assisted borohydride route using sodium borohydride. The presence of voids or pores in the as synthesized powders as well as the slower growth of particles during calcination process led to sustain its amorphous nature as well as loose porous structure up to 500 °C. Temperature mediated phase transformation from amorphous to crystalline nature of t-zirconium oxide took place at 600 °C and the t-zirconium oxide with porous structure was found to be stable up to 800 °C. In addition, the pore morphology of calcined (600 °C) t-zirconium oxide was found to be typically ink bottle-neck (H2-type) pores and it transformed to mixed ink bottle-neck and slit (H3 type) pores at 800 °C. Also, the mean pore diameter of 4.6 nm remains unchanged within a temperature range from 500 °C to 800 °C. Further, the t-phase of zirconium oxide transformed to mixed t- and m- phase with only slit type pores at 900 °C. The adequate surface area and pore volume along with nearly constant mean pore diameter of porous amorphous as well as crystalline t-zirconium oxide, developed through borohydride route may be suitable for different applications including adsorption of heavy metal ions for environmental application, shape-selective heterogeneous catalysis, ion exchange and proton conduction as well as gas-sensing application.

## Methods

Sodium borohydride (NaBH_4_), a novel H_2_ gas evolving reagent has been utilized to develop loose nature of zirconium hydroxide powders in the as-synthesized condition. Borohydride route via constant pH method was performed using aqueous Zr-salt (ZrOCl_2_·8 H_2_O) and aqueous NaBH_4_. First, aqueous solutions of 25 ml of 0.5 M ZrOCl_2_·8H_2_O (pH ~ 0.1) and 25 ml of 0.5 M NaBH_4_ (pH ~ 11) were prepared separately. While adding the acidic Zr-salt solution to a beaker containing of basic aqueous NaBH_4_, the pH of the solution decreases, so to maintain a constant pH environment of around 10.5, additional 0.5 M NaBH_4_ aqueous solution was added simultaneously along with Zr-salt solution. White precipitates were formed in the aqueous NaBH_4_ solution during the addition of both solution of Zr-salt and NaBH_4_ to the aqueous NaBH_4_. After completion of the reaction, the precipitate powders were filtered and thoroughly washed, dried, and calcined at different temperatures (500, 600 °C, 800 °C, 900 °C and 1000 °C) for 1 h. Phase analysis was performed by X-ray diffraction (XRD) using Cu K_α_ radiation. The crystallite size was calculated using Scherrer’s formula^58^ i.e. crystallite size (nm) = 0.9 λ /(β × Cos θ_B_), where λ is the wavelength of Cu K_α_ (0.154 nm), β is the Full width at half maximum (FWHM) and θ_B_ is the Bragg’s angle. β is expressed as (β_s_^2^ − β_std_^2^)^1/2^, where β_s_ is the FWHM of sample and β_std_ is the FWHM value (0.0511) for standard Si-wafer sample. The volume percentage of m and t-phase was determined using the formula 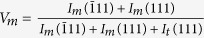 and 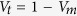 where I is the integrated intensity in each peak and m and t indicates monoclinic and tetragonal phase^38^. Particle morphology was studied using Transmission Electron Microscopy (TEM). The shape and size of various types of pores present in porous material are analyzed by Brunauer–Emmett–Teller (BET) method by using nitrogen adsorption–desorption hysteresis isotherms. The nature of the hysteresis loop can be divided into four different categories (H1, H2, H3 and H4) based on the IUPAC guideline^26^. According to IUPAC classification the H1 hysteresis are often associated with porous materials consisting of well-defined cylindrical-like pore with uniform sizes and shapes. Isotherms revealing type H2 hysteresis corresponds to channels with a pore mouth smaller than the pore body (this is the case of ink-bottle-shaped pores). The desorption branch for type H3 hysteresis contains a very wide distribution of pore size having slit like pores. Similarly, type H4 loops corresponds to limited amounts of mesopores limited by micropores. Surface area of zirconium oxide powders was determined using multi-points BET. Pore size distribution was analyzed using Barrett-Joyner-Halenda (BJH) method by considering BET-desorption behavior.

## Additional Information

**How to cite this article**: Nayak, N. B. and Nayak, B. B. Temperature-mediated phase transformation, pore geometry and pore hysteresis transformation of borohydride derived in-born porous zirconium hydroxide nanopowders. *Sci. Rep.* **6**, 26404; doi: 10.1038/srep26404 (2016).

## Figures and Tables

**Table 1:** Textural properties of borohydride derived porous nanopowders.

**Sample condition**	**Phase**	**Crystallite size** * **(nm) (XRD)**	**Particle size range (nm) (TEM)**	**Pore morphology**	**Pore hysteresis**	**Surface area (m**^**2**^**/g)**	**Range of pore size /Mean pore size (in nm)**	**Maximum Pore volume at mean pore size (cc/g)**
As-synthesized	A	–	–	Interconnected ink bottle-neck (major) + dumb-bell shaped pores (minor)	H2 type	182	3.6 to 15.7/3.6–4.6	0.21
Calcined at 500 °C	A	–	–	Interconnected ink bottle-neck + neck closing of a very few bottle-neck pores	H2 type	160	3.6 to 8.8/4.6	0.36
Calcined at 600 °C	t	7.5	10–20	Typically ink bottle-neck type pores	Typically H2 type	55	3.6 to 6.1/4.6	0.20
Calcined at 800 °C	t	18.8	20–40	Mixture of ink bottle-neck and slit type pores	H2 + H3 type	29	3.6 to 150/4.6	0.129
Calcined at 900 °C	t (78 vol%) m (22 vol%)	28.9	30–50	Typically lamellar or slit type pores	Typical H3 type	6	3.6 to 150/3.6	0.005
Calcined at 1000 °C	t (2 vol%) + m (98 vol%)	33.8	150–400	–	–	–	–	–

Note: A: amorphous; t: tetragonal phase of zirconium oxide; m: monoclinic phase of zirconium oxide.

^*^Crystallite size of t- zirconium oxide.
